# Strategic Characterization of Functional and Nutritional Traits in Yellow, Pink, and Black *Oxalis tuberosa* for Next-Generation Agricultural and Industrial Applications

**DOI:** 10.3390/foods15061004

**Published:** 2026-03-12

**Authors:** Franklin Oré Areche, Olivia Magaly Luque Vilca, Marino Bautista Vargas, Rafael Julian Malpartida Yapias, Alfonso Ruiz Rodríguez, Arcadio Sanchez Onofre, Severo Huaquipaco Encinas, Juan Alberto Julcahuanga Dominguez, Anyela Viviana Silva Guarnizo, Tania Jakeline Choque Rivera, Jhunior Marcía Fuentes

**Affiliations:** 1 Departamento Académico de Ingeniería Agroindustrial, Universidad Nacional de Huancavelica, Huancavelica 09001, Peru; alfonso.ruiz@unh.edu.pe (A.R.R.); severo.huaquipaco@unh.edu.pe (S.H.E.); 2 Departamento de Ingeniería de Procesos Industriales, Universidad Nacional de Juliaca, Juliaca 21101, Peru; oluque@unaj.edu.pe (O.M.L.V.); tj.choquer@unaj.edu.pe (T.J.C.R.); 3 Departamento Académico de Ciencias Agrarias, Universidad Nacional de Huancavelica, Huancavelica 09001, Peru; marino.bautista@unh.edu.pe (M.B.V.); arcadio.sanchez@unh.edu.pe (A.S.O.); 4 Departamento Académico de Ingeniería Agroindustrial, Universidad Nacional Autónoma Altoandina de Tarma, Tarma 12400, Peru; rmalpartida@unaat.edu.pe; 5 Departamento Académico de Ingeniería Pesquera, Universidad Nacional de Piura, Piura 20000, Peru; jjulcahuangad@unp.edu.pe (J.A.J.D.); asilvag@unp.edu.pe (A.V.S.G.); 6 Departamento Académico de Ingeniería de Procesos, Universidad Nacional de Agricultura, Catacamas 16201, Honduras; jmarcia@unag.edu.hn

**Keywords:** *Oxalis tuberosa*, yellow variety, functional properties, nutritional composition, agronomic performance

## Abstract

This study provides an integrated agronomic–functional–nutritional–bioactive characterization of three *Oxalis tuberosa* varieties (Yellow, Pink, and Black) cultivated under open-field conditions. Unlike previous studies that have typically examined isolated trait groups or single quality dimensions, this work simultaneously evaluates yield-related morphology, starch functional behavior, proximate composition, antioxidant activity, and pigment-associated color attributes within a unified experimental framework, enabling robust varietal comparison and application-oriented interpretation. The Yellow variety matured later (125 ± 2 days) and produced the highest total biomass (587 ± 32 g) and yield per plant (462 ± 28 g), with the longest tubers (8.7 ± 0.3 cm) and the greatest tuber number (12.1 ± 1.1 per plant). Functional assessments indicated that the Yellow variety exhibited superior swelling capacity (10.2 g/g) and solubility index (6.3%), together with the highest starch content (68.4 ± 2.1 g/100 g DW). Nutritional profiling further showed lower moisture and higher carbohydrate levels in the Yellow variety compared with the other varieties, supporting its suitability for food processing and agricultural production. In contrast, the Black variety showed the strongest antioxidant potential, with higher DPPH scavenging activity (46.2 ± 1.3%), total phenolics (5.9 ± 0.3 mg GAE/g DW), and flavonoids (2.3 ± 0.1 mg QE/g DW), consistent with its darker pigmentation and greater nutraceutical potential. The novelty of this study lies in its integrated, multi-trait comparison of oca varieties under the same open-field conditions with standardized agronomic management, allowing for the first simultaneous assessment of agronomic performance, starch functionality, nutritional quality, antioxidant capacity, and color attributes. Overall, these findings highlight the importance of varietal selection in determining agronomic performance, starch functionality, nutritional composition, and bioactive traits in *Oxalis tuberosa*, providing actionable evidence for targeted agricultural and industrial applications.

## 1. Introduction

*Oxalis tuberosa* (also referred to as oca) is a tuberous crop that is native to the Andean region of South America [1]. It is a valuable crop that is mainly cultivated due to its edible tubers that are a major source of food in most regions of the world, particularly in such countries as Peru and Bolivia [2]. Oca continues to be cultivated mainly through traditional farming systems, contributing to local food security and dietary diversity while maintaining substantial varietal diversity [3].

The species exhibits considerable phenotypic variability, expressed through differences in tuber color, shape, size, and growth behavior. Among the most widely recognized landraces are the Yellow, Pink, and Black varieties, which differ in tuber morphology, pigmentation, and developmental patterns [4]. The Yellow, Pink, and Black *Oxalis tuberosa* varieties were selected for this study because they represent three of the most widely cultivated and phenotypically contrasting farmer-maintained landraces in the central Andean region of Peru. These varieties are traditionally differentiated by tuber skin pigmentation, which has been associated in previous studies with differences in agronomic performance, starch composition, and accumulation of phenolic and pigment-related compounds. Empirical and ethnobotanical evidence suggests that yellow oca landraces are often preferred for higher yield and culinary versatility, whereas darker-colored varieties, particularly black oca, are associated with enhanced antioxidant and nutraceutical properties [4,5,6]. Pink varieties are frequently regarded as intermediate types, combining moderate agronomic performance with distinct sensory and color attributes. Selecting these three contrasting varieties therefore allows for a targeted evaluation of documented phenotypic and functional divergence while maintaining high agronomic relevance for Andean farming and application-oriented utilization. Such varietal diversity has direct implications for agronomic performance, nutritional composition, and technological functionality, underscoring the need for varietal-level characterization rather than generalized species-level descriptions [5,6].

From a nutritional and technological perspective, *Oxalis tuberosa* tubers are predominantly composed of starch, which constitutes the major carbohydrate fraction and represents the principal source of dietary energy [7]. Beyond its nutritional role, starch largely governs the functional behavior of oca in food systems, influencing hydration capacity, swelling behavior, viscosity development, gel formation, and stability during thermal processing [8,9]. Previous studies have shown that oca starch exhibits distinctive physicochemical characteristics, including high swelling capacity, variable solubility, and differential resistance to retrogradation [10,11]. Importantly, these properties can differ markedly among oca varieties due to genetic factors and tuber morphology, highlighting the importance of systematic varietal evaluation under open-field conditions with standardized management [12].

In parallel with its nutritional value, *Oxalis tuberosa* contains antinutritional compounds, particularly oxalic acid, which contributes to the characteristic acidic taste of fresh tubers [13]. Oxalic acid can reduce the bioavailability of essential minerals such as calcium and magnesium through the formation of insoluble complexes, thereby posing potential nutritional limitations [7]. Reported oxalate concentrations vary among oca varieties and are influenced by genetic background and environmental conditions, indicating that varietal selection plays a critical role in balancing nutritional benefits with antinutritional risks [14].

Despite growing interest in *O. tuberosa*, existing research remains fragmented, with most studies focusing on single trait domains such as agronomic performance, proximate composition, or phytochemical content often under heterogeneous environmental conditions [15]. Comparative studies integrating agronomic, functional, nutritional, antioxidant, and color traits across phenotypically distinct oca varieties cultivated under uniform conditions are scarce, limiting the ability to attribute observed differences to intrinsic varietal effects rather than environmental variability [16]. In particular, relationships among tuber morphology, starch-related functional properties, nutritional composition, pigment-linked color attributes, and antioxidant capacity remain poorly resolved.

The novelty of the present study lies in its integrated and comparative evaluation of three phenotypically distinct *O. tuberosa* varieties (Yellow, Pink, and Black) cultivated under open-field conditions. By jointly analyzing agronomic performance, tuber physical traits, starch-related functional properties, proximate nutritional composition, antioxidant activity, and CIELAB color parameters, this study provides a holistic characterization that links primary metabolism (yield and starch behavior) with secondary metabolism (phenolic content and pigmentation).

In addition to documenting varietal differences, this integrated framework enables a deeper examination of the physiological and biochemical bases underlying trait divergence among *O. tuberosa* varieties. By jointly analyzing yield-related morphology, starch-driven functional behavior, nutritional composition, antioxidant capacity, and pigment-linked color parameters, the study allows for an assessment of how primary metabolism (carbon allocation to starch and biomass) and secondary metabolism (phenolic and pigment synthesis) are coordinated or traded off at the varietal level [17]. Such an approach provides insight into the intrinsic relationships among agronomic productivity, functional performance, and bioactive potential that are not evident when traits are evaluated in isolation. Moreover, evaluating these traits under the same open-field conditions with standardized management reduces environmental and management-related variability, improving attribution of observed differences to intrinsic genetic and phenotypic variation among varieties [18]. This is particularly relevant for *O. tuberosa*, where traditional landraces are often studied across heterogeneous agroecological contexts, complicating comparison and limiting mechanistic interpretation. By integrating multivariate trait analysis with color-space and correlation-based approaches, the present study contributes to a more holistic understanding of how varietal identity shapes functional and nutritional outcomes.

Despite increasing interest in *Oxalis tuberosa* as a nutritionally and functionally valuable Andean tuber, a clear knowledge gap remains regarding how agronomic performance, starch-related functional properties, nutritional composition, antioxidant capacity, and color attributes are jointly expressed across distinct oca varieties grown under the same field conditions. Most previous studies have examined these traits in isolation, focused on single varieties, or were conducted under heterogeneous environmental and management contexts, limiting direct varietal comparison and obscuring potential trade-offs between primary metabolism (yield and starch accumulation) and secondary metabolism (phenolic and pigment synthesis). Consequently, there is a lack of integrated, application-oriented evidence linking varietal identity to coordinated agronomic, functional, and bioactive outcomes, which constrains informed varietal selection for targeted agricultural, food-processing, and nutraceutical applications.

Therefore, the objective of this study was to systematically compare the agronomic, functional, nutritional, antioxidant, and color-related traits of Yellow, Pink, and Black *Oxalis tuberosa* varieties cultivated under the same open-field conditions and standardized management practices, with the aim of identifying varietal strengths and trade-offs relevant to application-oriented utilization.

## 2. Materials and Methods

### 2.1. Study Location

The experiment was conducted in the Acobamba district within the province of Acobamba, region of Huancavelica, Peru, with the following geographical location; 12°50′30″ S latitude and 74°33′42.2″ W longitude with an altitude of 3417 m a.s.l. The experiment was conducted under open-field conditions in the Acobamba district, Huancavelica, Peru. Crops were grown under the prevailing local climatic conditions characteristic of the central Andean highlands.

No artificial control of temperature, humidity, or other environmental variables was applied. All varieties were grown simultaneously in the same field under identical agronomic management, ensuring that observed differences primarily reflect varietal effects under realistic field conditions for *Oxalis tuberosa* cultivation.

### 2.2. Plant Materials and Experimental Design

The study was performed on three types of *Oxalis tuberosa* (Yellow, Pink and Black). The tubers were bought from a local approved supplier and were selected safely to maintain homogenous size and status. The experiment was conducted in a randomized complete block design (RCBD) and each variety was repeated 3 times to attain statistical reliability [19]. The different varieties were allotted to different plots in each block to capture any form of differences that might exist in the environmental conditions, including the slight variation in soil texture or moisture content among the blocks. The overall experimental space occupied about 54 m^2^, with each plot occupying 2 m × 3 m (6 m^2^), which was sufficient to allow for the growth of plants, expansion of tubers, and ensuring that treatment interference was minimized. The plants had been planted in loamy soil with a pH of 6.5, which is suitable for the growing of tuber crops. Fertilization was applied uniformly using a balanced N–P–K (20–20–20) fertilizer delivered through irrigation water at a concentration of 10 g/L at weekly intervals. Irrigation was provided using a drip system, with watering frequency adjusted as needed to maintain adequate soil moisture for normal plant growth and tuber development under open-field conditions. Photoperiod was not experimentally manipulated. All varieties were exposed to the same natural daylight conditions prevailing at the study site during the growing season, ensuring a uniform light environment across treatments.

#### Germplasm Source and Varietal Identification

The three *Oxalis tuberosa* varieties evaluated in this study (Yellow, Pink, and Black) correspond to traditional farmer-maintained landraces that are commonly cultivated in the central Andean highlands of Peru and are primarily distinguished by tuber skin coloration and associated morphological traits. Planting material was obtained from a certified local germplasm supplier in the Huancavelica region, who maintains collections sourced from smallholder farming systems with long-standing cultivation histories. These landraces represent stable phenotypic groups rather than formally bred or genetically improved cultivars. Prior to planting, tubers were carefully selected to ensure uniformity in size, physiological maturity, and health status, and to confirm typical varietal coloration, thereby minimizing intra-varietal variability. No genetic modification or controlled breeding was applied, and the materials were propagated through traditional vegetative methods, reflecting their conservation and use under local agricultural practices.

The trial was conducted as an open-field experiment. Environmental conditions (temperature, relative humidity, and natural photoperiod) were not experimentally controlled; however, varietal comparisons were performed under standardized and uniform agronomic management (plot size, planting arrangement, irrigation, and fertilization applied equally across treatments).

### 2.3. Growth and Yield Parameters

To measure the physical characteristics of the plants, several growth and yield parameters were measured. Days to maturity were determined using a combination of aboveground indicators and periodic destructive sampling. Beginning approximately 20 days before the expected maturity window, plots were inspected weekly for foliage yellowing and partial senescence. At each assessment, three plants per plot were carefully uprooted to examine underground tubers for firmness (manual pressure), full skin set, and stable varietal pigmentation. Maturity was defined as the time point when at least 80% of sampled plants met these criteria. Plants not sampled were left undisturbed until final harvest [20]. The plant height was determined as the distance that covers the soil to the tallest part of the plant during harvest. Total biomass and harvest index were determined at final harvest using all remaining plants within each plot. Specifically, six plants per plot (eighteen plants per variety) were harvested after excluding plants removed during periodic destructive sampling for maturity assessment. Thus, growth and yield parameters were based on a total of 54 plants across all varieties. Total biomass was calculated as the combined fresh weight of leaves, stems, and tubers per plant, while harvest index was expressed as the ratio of tuber biomass to total aboveground and belowground biomass [21].

### 2.4. Tuber Characteristics

Tuber characteristics were evaluated using a randomized complete block design with three replications per variety (Yellow, Pink, and Black). At final harvest, five representative tubers were randomly selected from different plants within each replication, resulting in a total of fifteen tubers analyzed per variety. Tuber length and maximum diameter were measured individually using a digital caliper. The shape index (L/D), calculated as the ratio of tuber length to diameter, was used to describe tuber form, with higher values indicating more elongated tubers and lower values indicating rounder tubers. Mean values per replication were used for statistical analysis [20,22].

### 2.5. Functional Properties

Functional properties of *Oxalis tuberosa* tubers, including swelling capacity, solubility index, retrogradation resistance, and freeze–thaw stability, were determined following established starch functionality protocols widely used for tuber and root crops [9,23,24].

Swelling capacity was determined according to the method of Leach et al. [23]. Briefly, 5 g of dried tuber powder were suspended in 50 mL of distilled water and heated in a water bath at 85 °C for 30 min with intermittent stirring. The suspension was then cooled to room temperature and centrifuged at 3000× *g* for 15 min. Swelling capacity was calculated as grams of water absorbed per gram of dry sample.

The solubility index was measured using the same suspension. After centrifugation, an aliquot of the supernatant was dried at 105 °C to constant weight, and solubility was expressed as the percentage of soluble solids relative to the original dry sample mass [23,24].

Retrogradation resistance was evaluated by measuring syneresis following cold storage, as described by Hoover [9]. Starch pastes (10%, *w*/*v*) were prepared by heating the suspension to 95 °C for 10 min, cooled to room temperature, and stored at 4 °C for 24 h. The extent of water separation after centrifugation (3000× *g*, 15 min) was used as an indicator of retrogradation.

Freeze–thaw stability was assessed following the procedure described by Singh et al. [24]. Starch gels were subjected to three freeze–thaw cycles consisting of freezing at −20 °C for 24 h followed by thawing at 25 °C for 2 h. Syneresis after each cycle was recorded, and lower water separation was interpreted as higher freeze–thaw stability.

### 2.6. Nutritional Composition

Proximate composition (moisture, protein, fat, ash, and carbohydrates by difference) was determined using AOAC official methods [25], consistent with prior applications in Andean tubers [7]. The moisture content was determined by drying 5 g of tuber powder at 105 °C until constant weight. The protein content was assayed by the Kjeldahl method, employing digestion of the sample with sulfuric acid and distillation with a subsequent titration to measure the amount of nitrogen. The fat content was established through the Soxhlet method of extraction, in which 5 g of dried tuber sample was put in a Soxhlet extractor and extracted using petroleum ether. The percentage of ash was calculated by burning a known amount of tuber of dry powder in a muffle furnace at 550 °C for 5 h. Carbohydrate content was calculated by difference as 100 minus the sum of moisture, protein, fat, and ash contents. To determine energy content, it was computed as carbohydrate content × 4 + protein content × 4 + fat content × 9 and expressed per 100 g to obtain total energy (kcal/100 g).

The methods used for the determination of total phenolic content, flavonoid concentration, and antioxidant activity were adopted from well-established protocols previously applied to *Oxalis tuberosa* and other Andean tuber matrices. No, de novo methodological optimization was performed; however, extraction conditions (sample mass, solvent ratio, and reaction time) followed literature-validated procedures shown to be suitable for oca tubers. These protocols have been widely reported to provide reliable and reproducible estimates of phenolic content and antioxidant capacity in oca and comparable starch-rich tuber matrices, supporting their applicability to the present study.

### 2.7. Soil Properties

Several soil physical and chemical properties were analyzed to characterize the soil root-zone environment of the experimental plots. Soil bulk density was determined using the core sampling method, in which undisturbed soil cores (100 cm^3^) were collected at a depth of 0–20 cm, oven-dried at 105 °C to constant weight and expressed as dry mass per unit volume [26]. Soil penetration resistance (soil hardness) was measured in situ using a digital cone penetrometer, with readings taken at three points per plot and averaged [27].

Soil structural stability was assessed through measurements of resistance to deformation under applied force, expressed as relative soil firmness rather than food-based texture parameters. To avoid methodological ambiguity, terms such as “cohesiveness” and “chewiness” are used descriptively in Section 3 to indicate comparative soil resistance and pliability but are derived from penetrometer-based resistance metrics rather than food texture analysis [28].

Soil mineral composition was determined using Inductively Coupled Plasma Mass Spectrometry (ICP-MS) following acid digestion of air-dried, sieved soil samples (<2 mm). Concentrations of essential macro- and micronutrients, including calcium, potassium, magnesium, phosphorus, and iron, were quantified and expressed on a dry-weight basis [29,30].

Soil measurements were conducted to characterize plot-level conditions; however, soil properties were not experimentally manipulated and should therefore be interpreted as associative rather than causal with respect to varietal performance.

It is important to note that food texture properties of *Oxalis tuberosa* tubers were not evaluated in this study. Parameters such as hardness, cohesiveness, and chewiness refer exclusively to soil mechanical resistance indices derived from penetrometer-based measurements and are used descriptively to characterize soil structural conditions rather than tuber textural attributes. These measurements are not intended to represent sensory or instrumental food texture properties.

#### Replication and Sampling Scheme (Biological and Analytical Replicates)

The experiment followed a randomized complete block design (RCBD) with three biological replicates (blocks) per variety (Yellow, Pink, and Black), resulting in nine plots in total. Unless otherwise specified, plot-level means were used as the experimental unit for statistical analysis (*n* = 3 per variety). For agronomic and yield traits, six plants per plot were harvested at final harvest after excluding plants removed during periodic destructive sampling for maturity assessment (18 plants per variety; 54 plants total). Days to maturity were monitored weekly beginning ~20 days prior to expected maturity using three destructively sampled plants per plot per assessment; these plants were not included in final harvest measurements.

For tuber morphology (length, diameter, shape index), five representative tubers were randomly collected per plot at final harvest (15 tubers per variety). For starch content, functional properties (swelling capacity, solubility index, retrogradation, freeze–thaw stability), proximate composition, and antioxidant/phytochemical analyses, tubers collected within each plot were pooled, homogenized, and processed as one composite sample per plot; each assay was performed in triplicate as laboratory (technical) replicates, and the mean technical value was used as the plot value for statistical analysis (*n* = 3 plots per variety). Pooling of tubers within plots was applied to obtain representative plot-level compositional values and to minimize analytical variability. For soil properties, three subsamples per plot (0–20 cm) were collected and composited into one plot sample for ICP-MS mineral analysis, while bulk density and penetration resistance were measured at three points per plot and averaged to obtain one plot-level value.

### 2.8. Statistical Analysis

One-way analysis of variance (ANOVA) was used in the analysis of the obtained data to establish significant differences in the three varieties in all the measured parameters. Multiple comparisons were performed using Tukey’s HSD procedure [31]. All statistical tests were conducted with the help of the R software (version 4.3.1) [32] and the results were taken to be significant at *p* < 0.05. Pearson correlation analysis was employed to assess linear relationships among functional properties, among nutritional variables, and between CIELAB color parameters (L*, a*, b*) and antioxidant and phytochemical traits. Correlation matrices were used to facilitate interpretation of inter-trait relationships.

Pearson correlation analysis was applied to explore linear associations among functional, nutritional, colorimetric, and antioxidant variables because these parameters were continuous, approximately normally distributed at the plot level, and measured on comparable quantitative scales. Given the limited number of biological replicates (*n* = 3 per variety), correlation analyses were used in an exploratory manner to identify potential relationships rather than to establish definitive causal or predictive links. Accordingly, correlation coefficients were interpreted with caution, and emphasis was placed on the consistency and biological plausibility of observed patterns rather than on the magnitude of individual coefficients alone.

## 3. Result

Our study results showed that the significant varietal differences were observed in the structural and mineral properties of soils associated with the cultivation of the three *Oxalis tuberosa* varieties, indicating that soil–plant interactions varied among genotypes under open-field conditions. Bulk density differed significantly among all varieties, with the Yellow-associated soil showing the highest value, followed by Pink and Black. This gradient suggests progressively lower soil compaction from Yellow to Black, which may influence root penetration, water retention, and nutrient uptake. Soil mechanical properties and mineral availability further varied among treatments, highlighting the importance of considering soil–variety interactions when interpreting agronomic performance.

The magnitude of several statistically significant differences suggests not only analytical sensitivity but also potential biological and application-level relevance, particularly for traits directly linked to yield, starch functionality, and antioxidant capacity.

**Figure 1:**
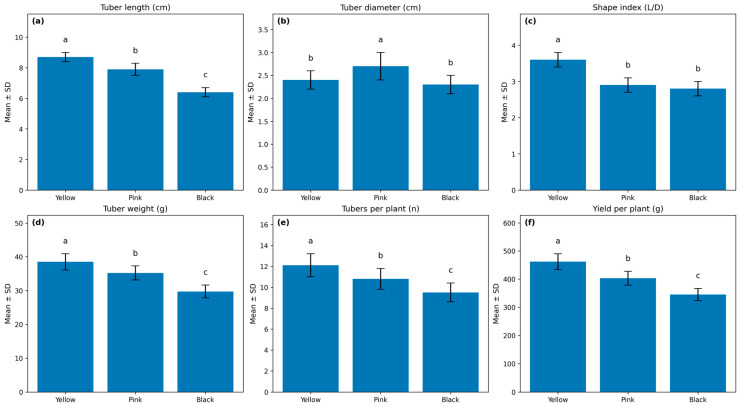
Physical and yield traits of *Oxalis tuberosa* varieties (Yellow, Pink, and Black): (**a**) tuber length, (**b**) tuber diameter, (**c**) shape index (L/D), (**d**) tuber weight, (**e**) tubers per plant, and (**f**) yield per plant. Bars represent mean ± SD (*n* = 3 replications). Different letters above bars indicate statistically significant differences among varieties based on one-way ANOVA followed by Tukey’s HSD test (*p* < 0.05).

The physical properties of *Oxalis tuberosa* varied significantly across the Yellow, Pink, and Black varieties, as illustrated in Figure 1. Clear quantitative differences were observed among the three *Oxalis tuberosa* varieties for all physical and yield traits. Tuber length ranged from 8.7 ± 0.3 cm in the Yellow variety to 6.4 ± 0.3 cm in the Black variety, with the Pink variety showing intermediate values (7.9 ± 0.4 cm) (Figure 1). This pattern indicates a substantially greater longitudinal tuber development in the Yellow variety. A contrasting trend was observed for tuber diameter, where the Pink variety exhibited the largest diameter (2.7 ± 0.3 cm), while Yellow (2.4 ± 0.2 cm) and Black (2.3 ± 0.2 cm) did not differ significantly, reflecting differences in tuber shape rather than size alone. The shape index (L/D) further highlighted these morphological distinctions. The Yellow variety showed the highest shape index (3.6 ± 0.2), indicating a more elongated tuber form, whereas Pink (2.9 ± 0.2) and Black (2.8 ± 0.2) produced significantly rounder tubers. These morphological differences were accompanied by marked differences in tuber mass and productivity. Mean tuber weight was highest in the Yellow variety (38.5 ± 2.4 g), followed by Pink (35.2 ± 2.1 g), and lowest in Black (29.7 ± 1.9 g).

Tuber number per plant showed a similar varietal ranking, with Yellow producing 12.1 ± 1.1 tubers per plant, significantly exceeding Pink (10.8 ± 1.0) and Black (9.5 ± 0.9). As a consequence, yield per plant differed substantially among varieties, reaching 462 ± 28 g in Yellow, compared with 403 ± 25 g in Pink and 345 ± 22 g in Black. Taken together, these values demonstrate that the Yellow variety consistently combines favorable tuber morphology with higher tuber number and mass, resulting in superior yield performance under the experimental conditions, while Pink and Black varieties display progressively lower yield-related traits.

### Functional Properties in Relation to Chemical Composition

**Figure 2:**
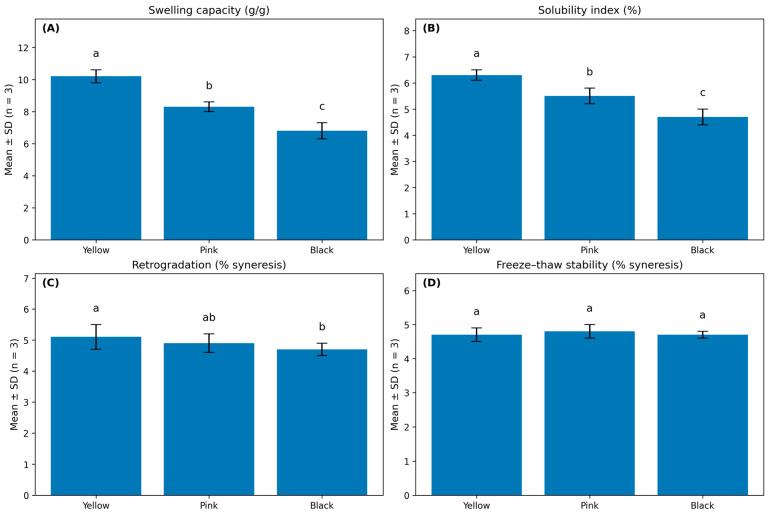
Functional properties of *Oxalis tuberosa* varieties (Yellow, Pink, and Black): (**A**) swelling capacity, (**B**) solubility index, (**C**) retrogradation (% syneresis after 24 h at 4 °C), and (**D**) freeze–thaw stability (% syneresis after three cycles). Bars represent mean ± SD (*n* = 3). Different letters indicate statistically significant differences among varieties based on one-way ANOVA followed by Tukey’s HSD test (*p* < 0.05).

Significant varietal differences were observed in the functional properties of *Oxalis tuberosa* tubers, reflecting underlying differences in chemical composition, particularly starch concentration and starch–phenolic interactions. The Yellow variety exhibited the highest swelling capacity (10.2 ± 0.4 g/g), which was significantly greater than that of the Pink (8.3 ± 0.3 g/g) and Black (6.8 ± 0.5 g/g) varieties (*p* < 0.05). Swelling capacity is largely governed by starch availability and granular organization; thus, the enhanced swelling behavior of the Yellow variety is consistent with its higher starch content (68.4 ± 2.1 g/100 g DW), which promotes greater water uptake and granule expansion during heating (Figure 2).

A similar varietal trend was observed for the solubility index. The Yellow variety showed the highest solubility (6.3 ± 0.2%), followed by the Pink (5.5 ± 0.3%) and Black (4.7 ± 0.3%) varieties, with all differences being statistically significant (p < 0.05). Higher solubility indicates increased leaching of soluble starch fractions and is typically associated with a less constrained starch matrix and greater polymer mobility. In contrast, the lower solubility observed in the Black variety suggests a denser starch structure and potential interactions between starch polymers and phenolic compounds that restrict solubilization (Figure 2).

Retrogradation behavior showed more moderate but meaningful varietal differences. Syneresis values were highest in the Yellow variety (5.1 ± 0.4%), intermediate in the Pink variety (4.9 ± 0.3%), and lowest in the Black variety (4.7 ± 0.2%), with the Yellow and Black varieties differing significantly (*p* < 0.05). Reduced retrogradation in the Black variety is consistent with its higher phenolic content, as phenolic compounds can interfere with amylose reassociation during cooling, thereby limiting recrystallization and water expulsion. In contrast, freeze–thaw stability did not differ significantly among varieties (*p* > 0.05). Syneresis values after three freeze–thaw cycles remained comparable, ranging from 4.7 ± 0.2% in the Yellow variety to 4.8 ± 0.2% in the Pink variety and 4.7 ± 0.1% in the Black variety. These results demonstrate clear varietal differences in swelling capacity, solubility, and retrogradation behavior among Yellow, Pink, and Black varieties (Figure 2).

Starch content differed significantly among the three *Oxalis tuberosa* varieties, indicating a clear varietal effect on carbohydrate storage and metabolic allocation. The Yellow variety exhibited the highest starch concentration, reflecting its greater capacity for carbon assimilation and reserve deposition in tuber tissues (Table 1). This elevated starch level is consistent with the superior agronomic performance observed for this variety, including higher biomass production and larger tuber dimensions, suggesting a strong linkage between sink strength and starch accumulation. The Pink variety showed an intermediate starch content, indicating moderate storage efficiency and carbohydrate partitioning relative to the other varieties. This pattern aligns with its intermediate agronomic and functional traits and suggests a balanced, but less intensive, accumulation of storage polysaccharides. In contrast, the Black variety presented the lowest starch concentration, which may be associated with a metabolic shift toward the synthesis of secondary metabolites rather than carbohydrate reserves. Starch content differed significantly among the three varieties, with the Yellow variety showing the highest values and the Black variety the lowest (Table 1).

The interrelationships among six nutritional parameters—moisture, protein, ash, fat, carbohydrates and energy—of Yellow, Pink and Black varieties of *Oxalis tuberosa* were explored using a comprehensive pair plot. The plot diagonal lines show kernel density histograms, and most of the traits have a relatively compact distribution within each variety. Yellow tubers had lower moisture levels and were compactly clustered in fat and ash structure, whereas the Pink and Black varieties were more varied particularly in moisture and protein. The off-diagonal scatterplots enabled the visual determination of linear relationships between parameters. There was a moderate negative relationship between moisture content and carbohydrate concentration, especially between the Yellow and Pink varieties. The energy content was found to be positively related to the carbohydrate content, particularly in the Yellow variety cluster, which supported the existing caloric value of carbohydrates in tuber crops. On the other hand, the protein content also showed weak or no apparent linear correlation with other macronutrients, which means that it varies independently. It is interesting to note that the scatter distributions indicate a partial clustering of the varieties according to the nutritional properties, with Yellow being a relatively well-coordinated cluster and the data points of the Black varieties being relatively spread, particularly, the dimensions of ash and protein. These trends highlight the possibility of discriminating tuber varieties on multivariate nutritional profiles and propose genetic or environmental components to maintain traits.

**Figure 3:**
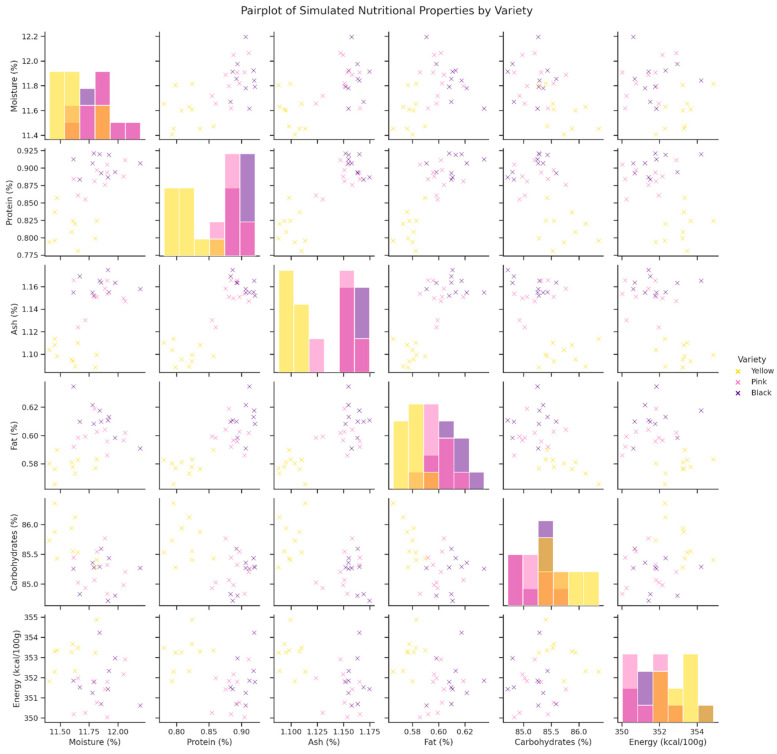
Pair plot showing the distribution and interrelationships of six nutritional traits across Yellow, Pink, and Black *Oxalis tuberosa* varieties. Histograms on the diagonal show the distribution of each variable, while off-diagonal panels display bivariate relationships. Colors indicate quinoa varieties: Yellow = yellow variety, Pink = pink variety, and Black = black variety. Note: The diagonal panels show the univariate distribution (histograms) of each nutritional parameter by variety, allowing comparison of central tendency, dispersion, and distribution overlap among groups. The off-diagonal panels present scatter plots illustrating pairwise relationships between variables. These panels enable visual assessment of potential correlations, clustering patterns, and varietal separation across nutritional traits. Clear color coding facilitates identification of group-specific trends, distribution overlap, and possible compositional differentiation among the three varieties.

The pair plot visualization (Figure 3) highlights coordinated and contrasting nutritional trait patterns among the three *Oxalis tuberosa* varieties. In particular, the clustering of Yellow samples reflects a coherent high-carbohydrate, high-energy profile with lower moisture variability, whereas the broader dispersion of Black samples indicates greater compositional heterogeneity, especially for protein and ash content. These patterns demonstrate that varietal differences are not limited to single nutritional parameters but emerge as multivariate profiles, supporting the conclusion that varietal identity shapes integrated nutritional outcomes rather than isolated traits.

Significant varietal differences were observed in the structural and mineral properties of soils associated with the cultivation of the three *Oxalis tuberosa* varieties, indicating that soil–plant interactions varied among genotypes under the open-field conditions. Bulk density differed significantly among all varieties, with the Yellow-associated soil showing the highest value (0.72 ± 0.01 g cm^−3^), followed by Pink (0.68 ± 0.02 g cm^−3^) and Black (0.65 ± 0.01 g cm^−3^) (Table 2). This gradient suggests progressively lower soil compaction from Yellow to Black, which may influence root penetration and water retention.

Soil mechanical properties also showed clear differences. Hardness was highest in the Yellow treatment (5.3 ± 0.2 N), intermediate in Pink (5.0 ± 0.3 N), and lowest in Black (4.4 ± 0.3 N), with Yellow differing significantly from Black. A similar trend was observed for cohesiveness, where Yellow soils exhibited greater internal resistance (0.62 ± 0.03) compared with Black soils (0.57 ± 0.03), while Pink showed intermediate behavior. Chewiness followed a distinct descending pattern, with Yellow (3.5 ± 0.2 N·mm) significantly exceeding Pink (3.1 ± 0.2 N·mm) and Black (2.9 ± 0.2 N·mm), indicating greater soil structural integrity and resistance to deformation in the Yellow plots (Table 3).

Mineral composition also varied significantly among varieties. Calcium content decreased from Yellow (41.2 ± 2.1 mg kg^−1^) to Pink (38.4 ± 1.9 mg kg^−1^) and Black (35.6 ± 2.0 mg kg^−1^), reflecting a clear varietal gradient. Iron content showed a contrasting pattern, with the highest concentration in Pink soils (1.4 ± 0.1 mg kg^−1^), while Yellow and Black did not differ significantly from each other. Potassium levels were highest in the Yellow treatment (330 ± 5 mg kg^−1^) and significantly lower in both Pink and Black soils, whereas phosphorus showed a moderate decline across varieties, with Yellow maintaining higher values than Black. Magnesium followed a similar trend, with Yellow soils exhibiting the highest concentration (28.6 ± 0.9 mg kg^−1^) and Pink and Black showing significantly lower but comparable levels (Table 2). Consequently, these results demonstrate that both soil structural characteristics and mineral availability differ significantly among the varietal treatments, with the Yellow variety generally associated with denser, mechanically stronger soils and higher concentrations of key macronutrients such as calcium, potassium, and magnesium. These soil conditions may contribute to the superior agronomic performance observed for the Yellow variety, highlighting the importance of considering soil–variety interactions when evaluating productivity and functional outcomes in *Oxalis tuberosa* cultivation.

**Figure 4:**
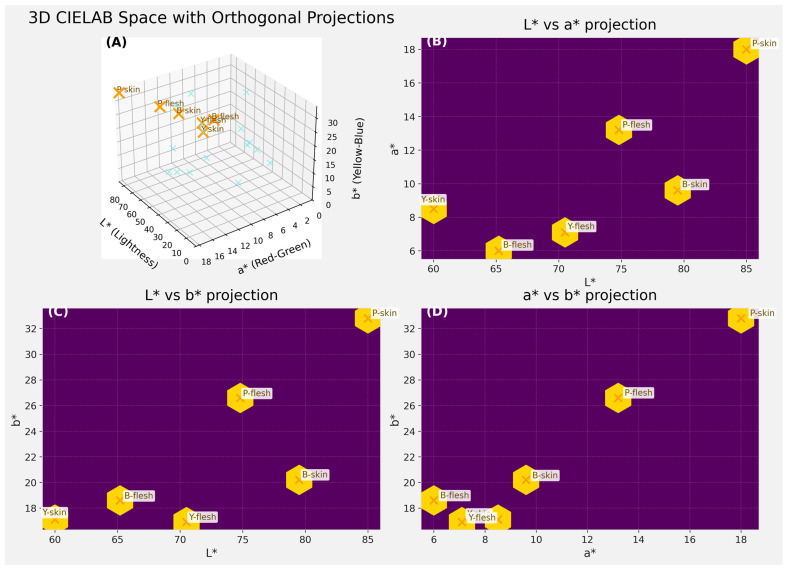
CIELAB visualization showing 3D color distribution and 2D chromatic projections (L*-a*, L*-b*, a*-b*) of Yellow, Pink, and Black *Oxalis tuberosa* varieties for both skin and flesh tissues. Note: The upper-left panel presents the three-dimensional CIELAB color space (L*, a*, b*), where L* represents lightness (0 = black, 100 = white), a* indicates the red–green axis (positive values = red direction), and b* represents the yellow–blue axis (positive values = yellow direction). Each point corresponds to the mean measured color coordinate of a specific sample (e.g., Yellow skin, Pink flesh, Black skin). (**A**) The “X” markers denote the centroid (mean L*, a*, b*) of each sample in the 3D space. The remaining panels show orthogonal two-dimensional projections of the same data: (**B**) L* vs. a*, (**C**) L* vs. b*, and (**D**) a* vs. b*. These projections allow clearer visualization of the relationships between pairs of chromatic parameters and facilitate comparison of lightness and chromatic intensity among samples. The hexagonal markers highlight the projected sample positions, while the “X” symbols represent their exact mean coordinates.

The advanced multi-panel CIELAB visualization (3D + 2D projections) illustrates the distinct color characteristics of the Yellow, Pink, and Black *Oxalis tuberosa* varieties in both skin and flesh tissues, revealing clear chromatic separation consistent with the overall phenotypic trends observed in our study (Figure 4 *In the 3D CIELAB space, pink samples form a distinct cluster in the region of higher* a and b* values, confirming their strong reddish–yellow pigmentation and greater overall chromatic intensity. Yellow samples occupy an intermediate position with moderate L*, a*, and b* coordinates, reflecting their lighter yellow appearance and relatively uniform skin–flesh color transition. By contrast, Black samples cluster toward lower L*, a*, and b* values, indicating darker, less saturated tones with reduced red and yellow components. The orthogonal projections (L*-a*, L*-b*, and a*-b*) further clarify these differences: Pink remains the most chromatically vivid variety, showing the highest a* and b* values; these color-space patterns are statistically supported by significant correlations between CIELAB parameters and antioxidant traits. Lightness (L*) was negatively correlated with total phenolic content and DPPH activity, while redness (a*) and yellowness (b*) showed strong positive correlations with phenolic concentration and antioxidant capacity (*p* < 0.05), confirming that darker and more intensely pigmented varieties exhibit higher levels of bioactive compounds (Table 3).

The multivariate CIELAB visualization (Figure 4) clarifies the relationship between pigmentation and antioxidant properties by showing distinct chromatic clustering of Yellow, Pink, and Black varieties across both skin and flesh tissues. The separation of Black samples toward lower L* values and their alignment with higher phenolic and antioxidant levels indicates that color parameters act as effective visual proxies for bioactive compound accumulation. This figure directly supports the conclusion that pigment-linked color traits are functionally associated with antioxidant capacity and can inform application-oriented varietal selection.

Antioxidants and phytochemical indicators differed significantly among the three *Oxalis tuberosa* varieties (*p* < 0.05). The Black variety showed the highest DPPH radical scavenging activity (46.2 ± 1.3%), followed by the Pink variety (41.5 ± 1.0%), while the Yellow variety exhibited the lowest value (34.8 ± 1.2%). Similar statistically significant varietal differences were observed for total phenolic content and flavonoid concentration, with the Black variety showing the highest levels (5.9 ± 0.3 mg GAE/g DW and 2.3 ± 0.1 mg QE/g DW, respectively), the Pink variety exhibiting intermediate values, and the Yellow variety showing the lowest concentrations (*p* < 0.05). A similar trend is observed in flavonoid content, with the Black variety containing the highest levels at 2.3 ± 0.1 mg QE/g DW, followed by the Pink variety at 1.9 ± 0.1 mg QE/g DW, and the Yellow variety at 1.5 ± 0.1 mg QE/g DW, which is the lowest among the three. Overall, the Black variety consistently demonstrates the highest antioxidant potential across all three measured parameters, indicating its superior antioxidant capacity compared to the Pink and Yellow varieties. The Black variety exhibited the highest DPPH scavenging activity, total phenolic content, and flavonoid concentration (Table 4).

**Figure 5:**
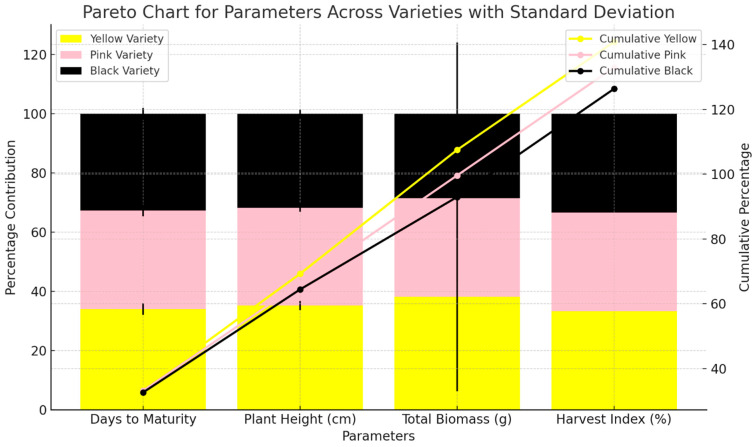
Agronomic performance of all three traits.

Regarding days to maturity, the results show that the Yellow variety takes the longest to mature, requiring 125 ± 2 days, followed by the Pink variety at 122 ± 2 days, and the Black variety, which matures the quickest in 120 ± 2 days (Figure 5). These differences indicate that the Black variety reaches full maturity slightly earlier than the other two varieties. In terms of plant height, the Yellow variety also stands out as the tallest, with an average height of 38.6 ± 1.5 cm. The Pink variety is slightly shorter at 36.2 ± 1.4 cm, while the Black variety is the shortest at 34.8 ± 1.3 cm. This suggests that the Yellow variety has a more robust growth in terms of vertical development compared to the Pink and Black varieties. The total biomass, which refers to the overall plant weight, shows that the Yellow variety again has the highest value, with 587 ± 32 g, followed by the Pink variety at 512 ± 28 g, and the Black variety, which has the lowest total biomass at 438 ± 24 g. These results indicate that the Yellow variety is more productive in terms of overall plant growth compared to the Pink and Black varieties. The harvest index, which measures the proportion of the plant’s biomass allocated to the harvestable portion (e.g., fruit or seeds), is quite similar across all varieties. The Yellow and Pink varieties have a harvest index of 78.7%, while the Black variety shows a slightly higher value of 78.8%. This suggests that although there are differences in total biomass, the efficiency of biomass allocation to harvestable parts is nearly identical among the varieties.

## 4. Discussion

The present study demonstrates clear and statistically significant varietal differences in agronomic, physical, functional, nutritional, and bioactive traits among the Yellow, Pink, and Black varieties of *Oxalis tuberosa*. These results confirm that varietal identity exerts a strong influence on tuber development, biochemical composition, and functional performance, reinforcing the importance of varietal-level evaluation for both agricultural productivity and downstream utilization. These findings confirm that varietal identity strongly shapes tuber development, biochemical composition, and functional behavior, highlighting the importance of varietal-level evaluation for both agricultural productivity and downstream utilization.

### 4.1. Physiological and Agronomic Basis of Varietal Differences

The superior agronomic performance of the Yellow variety, reflected in its greater tuber length (8.7 cm), higher biomass accumulation (587 ± 32 g), and increased tuber number per plant, suggests enhanced sink strength and assimilate allocation to storage organs. Longer and heavier tubers have been widely associated with improved carbon partitioning efficiency and overall plant vigor in root and tuber crops [20,33]. Genetic differences regulating tuber initiation, cell expansion, and storage parenchyma development likely underlie these patterns [34]. Campoverde Caicedo et al. [35] emphasized that farmer-maintained landraces exhibit distinct productivity, quality, and resilience traits shaped by long-term selection under Andean agroecological and biocultural systems. Similarly, Bonnave et al. [36] demonstrated that seed flow management and vegetative propagation strategies significantly structure the genetic diversity of oca, leading to stable varietal differentiation in morphological and functional characteristics. Together, these studies support the interpretation that the superior agronomic performance of specific oca varieties reflects intrinsic genetic differentiation reinforced by traditional cultivation systems rather than short-term environmental effects. Despite these differences in biomass and tuber morphology, harvest index values were remarkably similar among varieties, indicating that all three genotypes allocate a comparable proportion of total biomass to tubers. This suggests that yield differences are driven primarily by total biomass production rather than shifts in allocation efficiency, a pattern consistent with previous findings in tuber crops [37]. The strong agronomic performance of the Yellow variety is consistent with its higher starch accumulation, suggesting coordinated regulation of biomass production and carbohydrate storage that ultimately influences downstream functional properties.

### 4.2. Functional Properties and Starch-Related Mechanisms

Functional differences among *Oxalis tuberosa* varieties were closely linked to differences in chemical composition, particularly starch concentration and its molecular interactions with secondary metabolites. The Yellow variety exhibited markedly higher swelling capacity (10.2 g/g) and solubility index (6.3%), consistent with its significantly higher starch content (68.4 g/100 g DW). Although the varietal differences in starch content were statistically significant, they are also biologically meaningful; such differences are relevant for processing performance and formulation in starch-based food applications.

High starch availability promotes granule hydration and expansion during thermal treatment, especially when a greater proportion of amorphous polymer regions facilitate water penetration and solubilization [8,9]. These characteristics explain the superior hydration-related functional performance of the Yellow variety and support its suitability for applications requiring high water absorption and viscosity development [4,9]. Santacruz [38] reported that oca starch exhibits distinctive granule morphology, swelling behavior, and solubility patterns compared with other tuber crops, with variability largely attributed to differences in starch molecular organization and botanical origin. These findings support the interpretation that the enhanced swelling capacity and solubility observed in specific oca varieties are driven by intrinsic starch structural features rather than processing conditions alone. The proposed influence of phenolic compounds on starch swelling, solubility, and retrogradation in the present study is based on indirect evidence and established literature rather than on direct experimental characterization of starch–phenolic interactions. While our results show concurrent varietal patterns of lower starch functionality and higher phenolic content, the mechanistic interpretation that phenolics restrict starch hydration and reassociation is inferred from previously reported molecular interaction studies and not directly demonstrated through structural or binding analyses in this work [9].

In contrast, the Black variety exhibited lower swelling (6.8 g/g) and solubility (4.7%), together with reduced retrogradation (4.7% syneresis). This functional profile is consistent with its lower starch concentration and higher phenolic content. The Pink variety showed intermediate behavior across these functional parameters, reflecting a more balanced allocation between storage carbohydrates and secondary metabolites [39]. Freeze–thaw stability did not differ significantly among varieties, with syneresis values remaining within a narrow range (4.7–4.8%). This indicates that despite differences in starch concentration and phenolic composition, the starch–water networks formed after gelatinization were comparably stable across varieties during repeated freezing and thawing cycles, likely due to their shared botanical origin and similar granular architecture [40].

### 4.3. Nutritional Composition and Trait Interrelationships

Nutritional differences among varieties further highlight intrinsic trade-offs in metabolic allocation. The Yellow variety exhibited lower moisture content and higher carbohydrate and starch levels, supporting its superior energy density and storage stability. Lower moisture content is a critical determinant of shelf life and postharvest stability in tubers, reducing susceptibility to microbial spoilage and physiological deterioration [40]. The strong positive relationship between carbohydrate content and energy value observed in the Yellow variety reinforces its suitability for food security and processing applications where caloric yield is prioritized [41]. By contrast, protein content showed weak correlations with other macronutrients, suggesting independent genetic or physiological regulation. The narrower dispersion of nutritional traits in the Yellow variety indicates a more stable compositional profile, which is desirable for industrial applications requiring consistency in raw material quality.

### 4.4. Antioxidant Capacity and Pigmentation-Related Mechanisms

The Black variety exhibited the highest antioxidant capacity, total phenolic content, and flavonoid concentration, underscoring a contrasting functional specialization relative to the Yellow variety [42]. Darkly pigmented tubers are known to accumulate higher levels of anthocyanins and related polyphenols, which possess strong free-radical scavenging activity due to their conjugated aromatic structures and hydroxyl groups [7]. Reported variation in anthocyanin composition among oca landraces, particularly cyanidin- and delphinidin-based pigments, is strongly associated with antioxidant performance measured by DPPH assays [4]. Although individual phenolic compounds were not profiled in this study, the consistently higher phenolic and flavonoid contents of the Black variety provide a robust biochemical explanation for its superior antioxidant activity [43].

Beyond statistical significance, the higher phenolic and flavonoid concentrations observed in the Black variety are biologically relevant, as increases of this magnitude are associated with markedly enhanced radical-scavenging capacity and nutraceutical potential. From a practical perspective, these differences support varietal differentiation for health-oriented applications where antioxidant functionality, rather than caloric yield, is prioritized [42].

### 4.5. Color Attributes and Their Relationship with Antioxidant Properties

Colorimetric analysis revealed clear varietal differentiation in skin and flesh pigmentation of *Oxalis tuberosa*, as reflected by distinct CIELAB coordinates. The Black variety exhibited significantly lower L* values, indicating darker pigmentation, whereas the Pink variety showed higher a* and b* values, corresponding to more intense red and yellow chromatic components [44]. The Yellow variety displayed intermediate colour characteristics, consistent with its lighter visual appearance [4].

These colour differences were functionally linked to biochemical composition. Pearson correlation analysis demonstrated significant negative associations between L* values and both total phenolic content and DPPH radical scavenging activity, indicating that darker tubers possess higher antioxidant potential. Positive correlations were observed between a* and b* coordinates and antioxidant-related parameters, supporting the role of pigment intensity as an indicator of phenolic accumulation [2,7].

Although several correlations exhibited high coefficients, these relationships should be interpreted cautiously due to the small sample size, as strong correlations can be inflated under limited replication and primarily indicate directional associations consistent with known biochemical mechanisms rather than precise quantitative dependence.

The strong antioxidant performance of the Black variety can be explained by its higher accumulation of anthocyanins and other phenolic compounds, which are responsible for dark pigmentation and possess conjugated aromatic structures capable of efficient free-radical scavenging [7]. Previous studies on Andean tubers have demonstrated that cyanidin- and delphinidin-based anthocyanins are the dominant pigments in dark-colored oca landraces and are strongly associated with antioxidant activity measured by DPPH and related assays [2,4].

### 4.6. Soil–Variety Interactions and System-Level Implications

Significant differences in soil structural and mineral properties associated with varietal treatments suggest that soil–plant interactions may reinforce observed agronomic patterns. Higher bulk density and mechanical resistance in soils associated with the Yellow variety may enhance root anchorage and water retention, indirectly supporting higher biomass production [28]. Elevated concentrations of calcium, potassium, and magnesium further indicate improved nutrient availability that may contribute to enhanced growth performance [45]. However, as soil properties were characterized within the same experimental system, these differences should be interpreted as associative rather than strictly causal. Accordingly, soil mechanical parameters reported in this study are relevant for interpreting root growth conditions and potential influences on tuber development, but they should not be directly extrapolated to textural quality or processing behavior of oca tubers in food applications.

Although several soil parameters differed significantly among plots, these differences should be interpreted primarily in an associative context; their biological relevance lies in their potential to modulate root development and nutrient uptake rather than acting as direct drivers of varietal performance.

### 4.7. Limitations and Future Research Directions

Despite its integrative scope, this study has several limitations that should be acknowledged. First, while starch functional properties were characterized, detailed starch structural parameters such as amylose–amylopectin ratio, granule size distribution, crystallinity, and starch-bound phosphorus were not directly measured. These analyses would allow for a more mechanistic interpretation of the functional differences observed among varieties. Second, antioxidant capacity was assessed using global indicators (DPPH, total phenolics, flavonoids), but individual phenolic and anthocyanin compounds were not profiled, limiting insight into compound-specific contributions to bioactivity [46].

Additionally, the experiment was conducted under single-location open-field conditions with standardized management to isolate varietal effects; therefore, extrapolation to other agroecological zones and seasons should be made with caution. Future research should validate these varietal patterns under diverse agroecological conditions and incorporate molecular or metabolomic approaches to better resolve genotype–trait relationships. Integrating starch microstructure analysis, targeted phenolic profiling, and multi-environment trials would substantially strengthen the evidence base for application-oriented varietal selection. Nonetheless, the present work provides a robust comparative framework that advances understanding of *Oxalis tuberosa* as a multifunctional crop for next-generation agricultural, food, and nutraceutical systems [47].

Collectively, the multivariate visualizations (pair plots and color–antioxidant mappings) demonstrate that varietal differentiation in *Oxalis tuberosa* is best understood as coordinated trait syndromes encompassing agronomic performance, nutritional composition, functional behavior, and bioactive potential, thereby reinforcing the study’s central conclusion that targeted varietal selection requires an integrated, multi-trait perspective.

## 5. Conclusions

This study demonstrates statistically significant varietal differences in agronomic, functional, nutritional, and bioactive traits among Yellow, Pink, and Black *Oxalis tuberosa* cultivated under uniform open-field conditions. The Yellow variety consistently exhibited superior agronomic performance, including greater tuber length, higher biomass accumulation, and increased tuber number per plant, alongside higher starch content and enhanced starch-related functional properties such as swelling capacity and solubility. In contrast, the Black variety showed significantly higher antioxidant capacity, total phenolic content, and flavonoid levels, closely associated with its darker pigmentation. The Pink variety generally displayed intermediate characteristics across most evaluated parameters. These findings are directly supported by the experimental data and confirm that varietal identity strongly influences productivity, starch functionality, nutritional composition, and bioactive traits in *O. tuberosa*.

Based on these experimentally supported differences, the results suggest differentiated application potential among varieties. The high yield and starch functionality of the Yellow variety indicate suitability for food processing and starch-based industrial applications, whereas the elevated phenolic content and antioxidant capacity of the Black variety highlight promise for nutraceutical or health-oriented uses. These application-oriented implications represent potential future uses rather than direct performance testing and should be validated through targeted processing, sensory, and bioavailability studies. Overall, the integrated characterization provided here offers a robust evidence base to guide varietal selection, breeding strategies, and future research aimed at optimizing *Oxalis tuberosa* for agricultural, food, and nutraceutical systems.

## Figures and Tables

**Table 1:** Starch content of *Oxalis tuberosa* varieties.

Variety	Starch Content (g/100 g DW)
Yellow	68.4 ± 2.1 ^a^
Pink	63.7 ± 1.8 ^b^
Black	59.2 ± 2.0 ^c^

Values are mean ± SD (*n* = 3 replications). Different superscript letters within the column indicate significant differences among varieties (one-way ANOVA followed by Tukey’s HSD test, *p* < 0.05). DW = dry weight.

**Table 2:** Soil physical (bulk density, penetration resistance-derived indices) and mineral nutrient concentrations (ICP-MS) measured in plots cultivated with Yellow, Pink, and Black *Oxalis tuberosa* varieties.

Parameter	Yellow	Pink	Black
Bulk density (g cm^−3^)	0.72 ± 0.01 ^a^	0.68 ± 0.02 ^b^	0.65 ± 0.01 ^c^
Hardness (N)	5.3 ± 0.2 ^a^	5.0 ± 0.3 ^ab^	4.4 ± 0.3 ^b^
Cohesiveness	0.62 ± 0.03 ^a^	0.59 ± 0.04 ^ab^	0.57 ± 0.03 ^b^
Chewiness (N·mm)	3.5 ± 0.2 ^a^	3.1 ± 0.2 ^b^	2.9 ± 0.2 ^c^
Calcium (mg kg^−1^)	41.2 ± 2.1 ^a^	38.4 ± 1.9 ^b^	35.6 ± 2.0 ^c^
Iron (mg kg^−1^)	1.2 ± 0.1 ^b^	1.4 ± 0.1 ^a^	1.1 ± 0.1 ^b^
Potassium (mg kg^−1^)	330 ± 5 ^a^	312 ± 6 ^b^	305 ± 6 ^b^
Phosphorus (mg kg^−1^)	68.3 ± 2.0 ^a^	66.1 ± 1.8 ^ab^	64.4 ± 1.7 ^b^
Magnesium (mg kg^−1^)	28.6 ± 0.9 ^a^	27.2 ± 1.0 ^b^	26.5 ± 0.8 ^b^

Values are mean ± SD (*n* = 3). Different superscript letters within a row indicate statistically significant differences among varieties based on one-way ANOVA followed by Tukey’s HSD test (*p* < 0.05).

**Table 3:** Pearson correlations between CIELAB color parameters and antioxidant properties of *Oxalis tuberosa*.

Parameter	DPPH (%)	Total Phenolics	Flavonoids
L* (lightness)	−0.91 *	−0.94 *	−0.89 *
a* (redness)	0.93 *	0.96 **	0.92 *
b* (yellowness)	0.88 *	0.90 *	0.86 *

* Significant at *p* < 0.05;. Pearson correlation coefficients (r). ** significant at *p* < 0.01

**Table 4:** Antioxidant and phytochemical activity.

Parameter	Yellow Variety	Pink Variety	Black Variety
DPPH scavenging (%)	34.8 ± 1.2 ^c^	41.5 ± 1.0 ^b^	46.2 ± 1.3 ^a^
Total phenolics (mg GAE/g DW)	3.8 ± 0.2 ^c^	5.1 ± 0.2 ^b^	5.9 ± 0.3 ^a^
Flavonoids (mg QE/g DW)	1.5 ± 0.1 ^c^	1.9 ± 0.1 ^b^	2.3 ± 0.1 ^a^

Note: Values are mean ± SD (*n* = 3). Different superscript letters within a row indicate statistically significant differences among varieties based on one-way ANOVA followed by Tukey’s HSD test (*p* < 0.05).

## Data Availability

The original contributions presented in this study are included in the article. Further inquiries can be directed to the corresponding author.
